# Rodent Models and Behavioral Outcomes of Cervical Spinal Cord Injury

**DOI:** 10.4172/2165-7939.S4-001

**Published:** 2013-07-27

**Authors:** Sydney A. Geissler, Christine E. Schmidt, Timothy Schallert

**Affiliations:** 1 Department of Biomedical Engineering, The University of Texas, Austin, TX, USA; 2 Professor, J Crayton Pruitt Family Department of Biomedical Engineering, University of Florida, Gainesville, FL, USA; 3 Professor, Department of Psychology, The University of Texas, Austin, TX, USA; 4 Professor, Department of Neuroscience, The University of Texas, Austin, TX, USA

**Keywords:** Cervical spinal cord injury, Behavioral assessment, Rodent models, Regeneration, Spinal cord injury surgical models, Behavioral deficit, Functional recovery, Forelimb function

## Abstract

Rodent spinal cord injury (SCI) models have been developed to examine functional and physiological deficits after spinal cord injury with the hope that these models will elucidate information about human SCI. Models are needed to examine possible treatments and to understand histopathology after SCI; however, they should be considered carefully and chosen based on the goals of the study being performed. Contusion, compression, transection, and other models exist and have the potential to reveal important information about SCI that may be related to human SCI and the outcomes of treatment and timing of intervention.

## Introduction

Rodent models are designed to help predict functional outcomes of neurological disorders and injuries. Many of the behavioral outcomes appear to parallel clinical symptoms observed in human patients to a remarkable degree. About 11,000 people in the United States sustain SCI, leaving over 200,000 people living with debilitating levels of chronic paralysis. Further, 62% of these injuries are at the cervical level (http://www.uab.edu/medicine/sci/). Unilateral cervical spinal rodent models are desperately needed to assess the potential of treatments to help patients regain function. Using different injury models and sensitive behavioral tests that target the site and extent of injury, treatments aimed at improving functional recovery can be more adequately evaluated. Some models are considered more clinically relevant than others; however, it is important to remember that human SCI is very complex and many different injury types occur in human patients.

Understanding the strengths and limitations of the models will allow more relevant analysis of the injury, behavioral sequel, and therapeutic approaches.

## Injury Models of SCI

Contusion and compression injuries are the most common forms of SCI in humans. Researchers can create these injury types using balloon compression, spring-loaded clips, and computer-controlled impact. These devices can be controlled for force of impact and dwell time that the force is applied. Each model has strengths and weaknesses (Table 1). This allows researchers to tailor the injury to their specific goals.

Balloon compression models are advantageous because they can precisely control the time and force of the compression and can be performed percutaneously, without a laminectomy. This model mimics a burst intervertebral disc injury in humans. These injuries are often not considered relevant because the decompression occurs much earlier than would occur in clinical cases. Spring-loaded clip models also allow good control of the time and force of the injury and are easy to perform with precise placement. This is one of the most inexpensive options for compression or contusion injury models. However, clip compression models require extensive decompression of the spinal canal which may cause further damage or inflammation. Compression injuries can also be performed with modified surgical forceps to create a reproducible functional deficit, and are more commonly used in a thoracic injury model [1,2]. Static weight compression is another model in which a known weight is placed gently on the spinal cord and held there for a controlled period of time [3]; because there is less acute damage, this model is not as clinically comparable, but still serves an important role to assess the damage of compression alone. These models are especially useful to assess the most optimal decompression time after injury [4] and have helped change the treatment of human patients after SCI. Indeed, prior to 1997 surgery was not performed as quickly as possible on patients in fear of doing further harm, but currently, patients are rushed into surgery to remove pressure from the spinal cord to prevent further damage [5].

Computer-controlled impactor injury models are generally recognized as the most clinically relevant models [6], and are well suited for measuring functional deficit. There are many different devices available for this model: (1) the MASCIS device uses a weight drop technique with different heights to control the severity of the lesion and has been well characterized for thoracic SCI [7], (2) the OSU device controls the displacement of the head to control severity of the lesion [8,9], (3) the PinPoint device from Hatteras that allows control over velocity and displacement which was originally described for thoracic injury [10], and (4) the Infinite Horizon Impactor controls applied force to control the severity of the lesion, which has been thoroughly characterized for thoracic [11] and cervical SCI [12]. These approaches can all be used to create reproducible lesions; however, the necessary equipment can be expensive to purchase and the techniques require extensive training for proficiency. These models have been used to examine functional deficits and correlate them to histopathology [12], to examine demyelination [13], and as a means to assess biomaterial delivery [14] and cell delivery [15] for repair and regeneration.

Transection models are considered less clinically relevant; however, they allow researchers to be precise in the tracts affected, and allow them to tailor their behavioral tests to assess the functional recovery most accurately. Transection models are usually performed with precision to remove specific tracts or areas of tissue. Dorsal hemisection [16], dorsal quadrant [17], lateral hemisection [18], dorsal column transection [16], and other models have been performed. These models are especially useful for assessing axon regeneration because the amount of axon sparing should be minimal or controllable, especially if a complete transection is performed. Researchers have used transection models to examine multiple treatments, including biomaterials [19], drugs or growth factors [20], and stem cell transplantation [21]. If researchers wanted to compare and assess the ability of various treatment options to support regeneration of the rubrospinal tract, then a quadrant or lateral hemisection can be very useful to guarantee that no axon sparing occurs. SCI lateral hemisection models include a lesion that removes half of the tissue laterally and is generally performed manually by the experimenter. This model has similar deficits as the human Brown-Sequard syndrome, in which half of the body loses function, whereas the other half of the body retains function [22]. Dislocation [23], and ischemia [24], has also been modeled anatomically and behaviorally.

## Behavioral Tests

Researchers also need to determine which behavioral tests are most relevant for their species, model, and goals. Just as models will show different deficits based on which tracts are affected, behavioral tests reveal specific functional deficits? For example, if a model damages the corticospinal tract only, the animal’s locomotion should not be seriously affected, so behavioral tests examining locomotion should not be the only ones used. Similarly, physical therapists and physicians examine human patients using the American Spinal Injury Association (ASIA) scale to look at specific deficits. This scale is used to determine the extent and level of injury for patients (http://www.asia-spinalinjury.org/). Patients are rated on specific functions that are associated with levels of the spinal cord (e.g., C5 is associated with bicep strength) and given a score of 0–5 where 0 is complete paralysis, and 5 is normal function. Scores of all of the tested functions are then combined to give them an overall score from A–E where A is complete paralysis (including sensory function) and E is normal function (http://www.asia-spinalinjury.org/elearning/ISNCSCI_Exam_Sheet_r4.pdf).

Vibrissae-elicited placing is a commonly used test to examine the sensorimotor function after spinal cord injury in rats [18,25]. This test involves stimulating the whiskers on a table edge to elicit a motor response of the animal placing its forepaw onto the table to gain stability (http://homepage.psy.utexas.edu/homepage/group/schallertlab/Et-1%20placing.mpg). A graded score of 0–4 is assigned based on the extent of movement of the limb being tested where 0 is complete paralysis, and 4 is normal function. This test can indicate the integrity of motor and sensory tracts. This test eliminates the possibility of a reflex response being elicited; therefore, this test can reveal that a motor tract is intact along the distance it travels. Based on the level of function, this test may also reveal the extent of damage to the cross-sectional area of the motor tract.

Contact placing is similar to vibrissae-elicited placing and involves a light touch to the paw from which the motion is being elicited; this test has been performed on rats [26] but is difficult to perform on mice. This involves a light touch that does not signal the pressure sensory nerves in the skin to elicit a response, and the experimenter must be careful to not move the limb or any joints during testing. This test can show if there is a reflex; however, it will not differentiate between a reflex and a complete tract connection, where the tactile information made it to the brain prior to eliciting the response. This test is similar to the tests performed for the ASIA scale used for SCI patients to determine if they can feel light touch. In human patients, they are asked to tell the doctor if they feel the light touch; however, with animals, that is not possible, so this test incorporates motor movement as the signal to show that the light touch is perceived.

Proprioceptive placing indicates whether the reflex arc is still present. If the animals are able to respond to light touch, this test will not work because they will respond before there is motion of the limb. The test has been performed on rats [26] but is difficult to perform in mice. By stretching the tendon as a joint is extended, a reflex response is elicited. This is similar to the reflex tests performed for the ASIA test in human SCI patients.

Limb use asymmetry during vertical-lateral exploration in a cylinder (20 cm diameter, 30 cm height for rats; 11 cm diameter, 20 cm height for mice) is an easy, inexpensive test to determine whether the animal has a preference in its limb use; this test has been used extensively in rodents [25,27–30]. The cylinder test involves allowing the animals to move naturally and vertically explore the walls around them while the experimenter records which limbs are used for weight supported stepping on the wall (http://homepage.psy.utexas.edu/homepage/group/schallertlab/CylinderTest.mpg). This allows a clear determination of sidedness in natural function. This would allow experimenters to determine which side of the animal was more affected by the injury, and in the case of unilateral injuries, it allows the experimenters to determine the level of use of the ipsilesional vs. contralesional limb.

Swim tests can reveal functional deficits in forelimbs and hindlimbs. Normal rodent behavior when swimming to an escape platform is to hold both forelimbs forward under their chin in a planing position while stroking with the hindlimbs [31]. With a deficit in the forelimbs, an animal will not be able to plane their forepaws. This can result in one limb dragging. This test is very reliable for cervical SCI injuries to determine presence of forepaw planing behavior, the angle of a limb that is not planing, and deficits in hindlimb stroking motions [30].

Open-field locomotion is used to assess the animal during normal functional movement. This involves observing and analyzing the position and weight support on individual forelimbs during uninterrupted locomotion. These tests are sensitive to the amount of limb use when the animals are behaving naturally. One well standardized open field test is the Basso, Beattie, and Bresnahan (BBB) test. Although, it is better suited for thoracic injuries, it has been used in low cervical injuries extensively as well [32]. There are a few open-field locomotion tests, including the FLS [34], FLAS [35], and one developed by a group at the University of Provence in Marseille, France [36] that are more specifically designed for cervical injuries. Each test provides a score for different behaviors that are sequential categories of forelimb function, including weight support on the limbs, paw position and orientation, and swinging motion. These tests observe animals in a similar situation to human locomotion because they do not use forced motion of the ipsilesional limb and allow the animal to compensate for the injury the way they usually compensate while moving on their own. Compensation occurs frequently in rodents and humans to increase speed, efficiency, or accuracy of a movement but does not exercise or demonstrate the functional ability of the ipsilesional limb.

Forepaw dexterity tests show fine motor movements and reveal paw strength and function. Animals can use their paws differently based on the model and the tracts affected. Similarly to human deficits, some animals cannot grip an object, and some cannot release their grip on an object. Some can move individual digits while others have only moderate wrist or shoulder function. A few pasta tests have been developed to reveal these differences. The Jones and Whishaw groups have developed a test to examine individual digit function [37,38], while Schmidt group uses a similar pasta type to examine overall forelimb function [18]. Other handling tests use different types of foods, including cereal as the IBB test [33], peanuts, and grapes among other types [39] to examine functional deficits. Time to eat the object, forepaw use, and individual digit motion are all examined in these tests. Improvement can be quantified as the animals increase use of the ipsilesional limb. These are similar to human SCI patients’ abilities to grip and manipulate objects. The ASIA exam includes finger strength and abduction assessment to determine injury level (http://www.asia-spinalinjury.org/).

Forced motion tests have been developed to assess the functional abilities of each forelimb while preventing compensation with the other limb. The postural instability test (PIT) (http://homepage.psy.utexas.edu/homepage/group/schallertlab/PIT1.mpg) and a forelimb alternation test (http://homepage.psy.utexas.edu/homepage/group/schallertlab/alt.v.nonalt_mpg.mpg) have been used to examine forelimb function during forced forward motion [18]. These tests are scored by determining the distance before motion is elicited while the animal is moved forward with only forelimb support, showing improvement as the score aligns with the pre-op distance. These tests allow researchers to examine an animal’s ability to use the limbs while removing the chance for compensation with the other limb, giving the experimenter an understanding of actual functional abilities whether or not the animal has been compensating for that same motion. This is more comparable to clinical tests, as most of the tests performed on patients examine a patient’s ability when they consciously attempt to make individual movements.

This is by no means a comprehensive review, and many other behavioral tests exist such as the Montoya staircase food-retrieval [12, 25], grooming [12,27], automated walkway [12,27], inclined plane [32,40–42], and horizontal ladder [27] tests. Each of these tests can reveal specific functional recovery and researchers should consider which tests are most relevant for their goals and expectations. Further, we did not describe many sensory tests here and these should also be considered. The dot patch removal test [16,25,30], Von Frey hairs [42], and heat test [43] can all elicit responses to sensory input and may provide insight to hyperalgesia [44].

## Considerations

When determining which animal model and behavioral tests to use, researchers have many factors to consider, including goals, species-specific differences, cost, and analysis time and ability. The detail that can be attained using many behavioral tests should not outweigh the importance of standardized measurement between research groups with similar goals. Comparability of the models and assessment is critical to translate treatment to the clinic. Further, it is important to recognize the importance of planning. Researchers should choose models to meet their goals rather than performing a barrage of tests and reporting the most significant findings.

The therapeutic goals should match the damage expected by the model [7]. For example, if a treatment is designed to specifically connect rubrospinal tract neurons, a moderate midline contusion injury will not suffice to cause much damage to that region. The region of interest also needs to be chosen wisely to achieve specific deficits and to allow for recovery.

To determine which species and strain to use for the study, researchers must also consider what their goals are. Rats exhibit similar physiological changes to humans after SCI in that a cavity forms within the area. Mice do not exhibit this physiology; however, genetically controlling mice is much easier and physiological changes can be examined more easily using specific mouse strains. Such differences in rodents can provide powerful analysis if the correct animal model is chosen. Researchers have also used larger animals such as cats [44,45], dogs [46], and primates [47], or smaller animals, such as eels [48], zebrafish [49], and lamprey [50]. The regenerative capacity in each species varies and provides a platform for examining different characteristics of injury based on the experiment.

With each of these considerations also comes cost. Different injury models may be much more expensive to implement. The Infinite Horizon Impact or is much more expensive than a set of aneurysm clips, and a transection model can be performed with standard surgical instruments, making it even less expensive. Further, the cost of the species used, and the cost of post-operative animal care should be considered.

Anatomical analysis of the histopathology can be time consuming. Tract tracing can elucidate which specific tracts were spared or regenerated after injury, and specific staining can inform researchers about myelin damage and glial responses. However, physiological examination does not speak to the function of the tissue. Electrophysiological assessment would be very valuable to determine functionality of specific regions; however, it is very difficult to carry out. Behavioral assessment should be performed to assess functional deficit and recovery in combination with histological assessment. Behavioral assessment can elucidate the influence of the damage and repair of the tissue on overall function. Different models can make some analysis more easily performed or relevant; for example, transection models can be advantageous to eliminate axon sparing, but sprouting and axon sparing are more relevant in a contusion model. Transection models create a space for placement of pre-formed biomaterials, but if a treatment is injectable, a contusion model may be chosen to more closely mimic clinical injuries.

It is important to consider the leading causes of morbidity and mortality among rodent test subjects. Autophagia is common for all injury models [51,52]. Respiratory tract infections and decubitus are also concerns for researchers working with cervical spinal cord injury [53]. Urinary tract infections are less prevalent with cervical than with thoracic SCI models but should still be monitored [53].

## Conclusion

Rodent models cannot predict exactly how human patients respond to SCI physiologically or functionally; however, they provide an early assessment platform for treatment and physiological damage after SCI. Human SCI is very complex and there is an appreciation among researchers that the models cannot mimic the injuries perfectly, nor can they expect all behavioral symptoms observed in humans to have correlates in animals, or vice versa. It is important to examine behavioral deficits in rodents, even without a human correlate, to determine if there is a functional change. Although humans may not show an obvious vibrissae-elicited placing reaction, this test can reveal a loss, and a return, of sensory and motor communication between the brain and spinal cord. An animal’s ability to perform in different tasks can reveal specific tracts that are regenerating, even if they are not in the same location in the human spinal cord. This can reveal a promising treatment that can then be tested further on higher species or directly on humans in a hope to help with recovery. It must be kept in mind that treatments that rescue neurons from degeneration may sometimes have an adverse effect on functional outcome [54].

Many surgical models exist to study SCI in rodents; however, it is important to assess the goals and expectations of the experiment before choosing a model. Similarly, behavioral tests need to be chosen carefully to ensure that a researcher is examining a relevant function. It can be tempting to use a barrage of tests and use the tests that show the most promising or significant results; however, this is not a scientifically sound way to determine the recovery after SCI. Each aspect of a study should be planned before the experiment is begun and a pilot study is recommended to provide insight into what is expected from each model and treatment to minimize animal use and unnecessary experimental time during the experiment.

## Figures and Tables

**Table 1:** Pros and cons of surgical models. Differences in surgical models allow researchers to choose the surgical model to best study their goals.

Injury Model	Pros	Cons	Clinical Correlates
**Compression**			
Balloon Spring-loaded clip Modified forceps Static weight	Control of time of application Control of force of application Useful to assess decompression time Inexpensive	Decompression occurs much earlier than in clinical settings Extensive lamina removal causing extensive decompression	Intervertebral disc burst injury Determine optimal decompression time
**Computer-controlled impactor contusion**			
MASCIC OSU Infinite Horizon PinPoint	Reproducible lesions Useful to assess demyelination and axon sparing Considered most clinically relevant model	Expensive	Considered most clinically relevant model
**Transection**			
Dorsal hemisection Dorsal quadrant Lateral hemisection Dorsal column	Useful to assess axonal regeneration Inexpensive	Considered less clinically relevant	Stab or shooting injury Brown-Sequard syndrome
